# Supplementary material for: Video-rate high-precision time-frequency multiplexed 3D coherent ranging
Source: Nat Commun. 2022 Mar 29;13:1476. doi: 10.1038/s41467-022-29177-9 (PMC8964719; doi:10.1038/s41467-022-29177-9)
Supplement: Supplementary file 2 — Supplementary Information [file 41467_2022_29177_MOESM2_ESM.docx]

**Video-rate high-precision time-frequency multiplexed 3D coherent ranging**

Ruobing Qian^1^, Kevin C. Zhou^1^, Jingkai Zhang^1^, Christian Viehland^1^, Al-Hafeez Dhalla^1^ & Joseph A. Izatt^1,2,*^

^1^ Department of Biomedical Engineering, Duke University, Durham, NC, USA, 27708, USA

^2^ Department of Ophthalmology, Duke University Medical Center, Durham, North Carolina 27710, USA

*Correspondence author: [joseph.izatt@duke.edu](mailto:joseph.izatt@duke.edu)

**Supplement Information**

**Lateral resolution along the grating axis**

To study the lateral resolution of our system along the grating axis, which is determined by the sum of PSFs of all the wavelengths within the same spectral window, we performed the following modeling. In other words, it is the convolution between the optical PSF and the STFT window.

Given that the Airy radius of our PSF (0.61$\lambda/NA$) at the focal plane is about 890 µm, we can approximate the PSF as a gaussian function with standard deviation σ$\approx0.21\lambda/NA$=302.6 µm ^1^. We simulated 200 PSFs evenly spaced within the spectral window, which match the number of spectral sampling points of each window. Here in Supplement Fig.1(a), we showed five representative normalized PSFs within the same spectral window, including the first and the last PSF, with the peak of the first PSF (-445 µm) located at 890 µm away from the last PSF (+445 µm). We then calculated the sum of all 200 PSFs and normalized its maximum value. The plot of the normalized sum of PSFs is shown in Supplement Fig.1(b) along with the single PSF. We calculated the standard deviation of the new summed PSF and converted it to the Airy radius. The Airy radius of the new summed PSF was found to be 1240 µm.


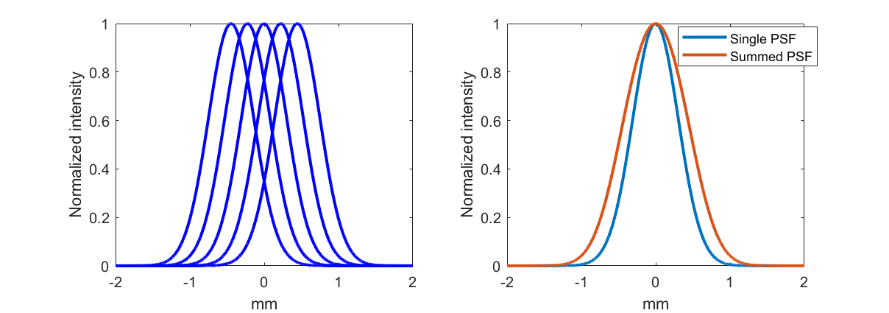


**Supplementary Figure 1: Characterization of lateral resolution along the grating axis.** **(a)** Five representative normalized PSFs within the same spectral window; **(b)** the normalized 200 summed PSFs within the same spectral window along with the single PSF

**Noise removal in the processed depth map**

The processed depth map of the mannequin head is shown in Supplement Fig. 2(a). A lower threshold value compared to the metal and cup samples was applied in order to localize most of the reflectors inside the face, and the stripe noise due to imperfect removal of invalid points during the transitions between subintervals of a laser sweep can be observed ^2^, as well as the depths at some lateral positions within the face are not successfully located. First, we applied a 3*3 median filter and a Sobel filter ^3^ to detect the pixels with large gradient magnitudes (Supplement Fig. 2(b)). We then removed the depths of all the pixels that had gradient magnitudes above a predefined threshold value from the original depth map (Supplement Fig. 2(c)). Some major vertical stripe noise was successfully removed from the depth map, while all the localized depths within the sample were maintained. Finally, we applied a 3*3 median filter to remove the rest background noise and estimate the depths within the samples that were not successfully localized in the original depth map. The final processed depth map of the mannequin head is shown in Supplement Fig. 2(d).


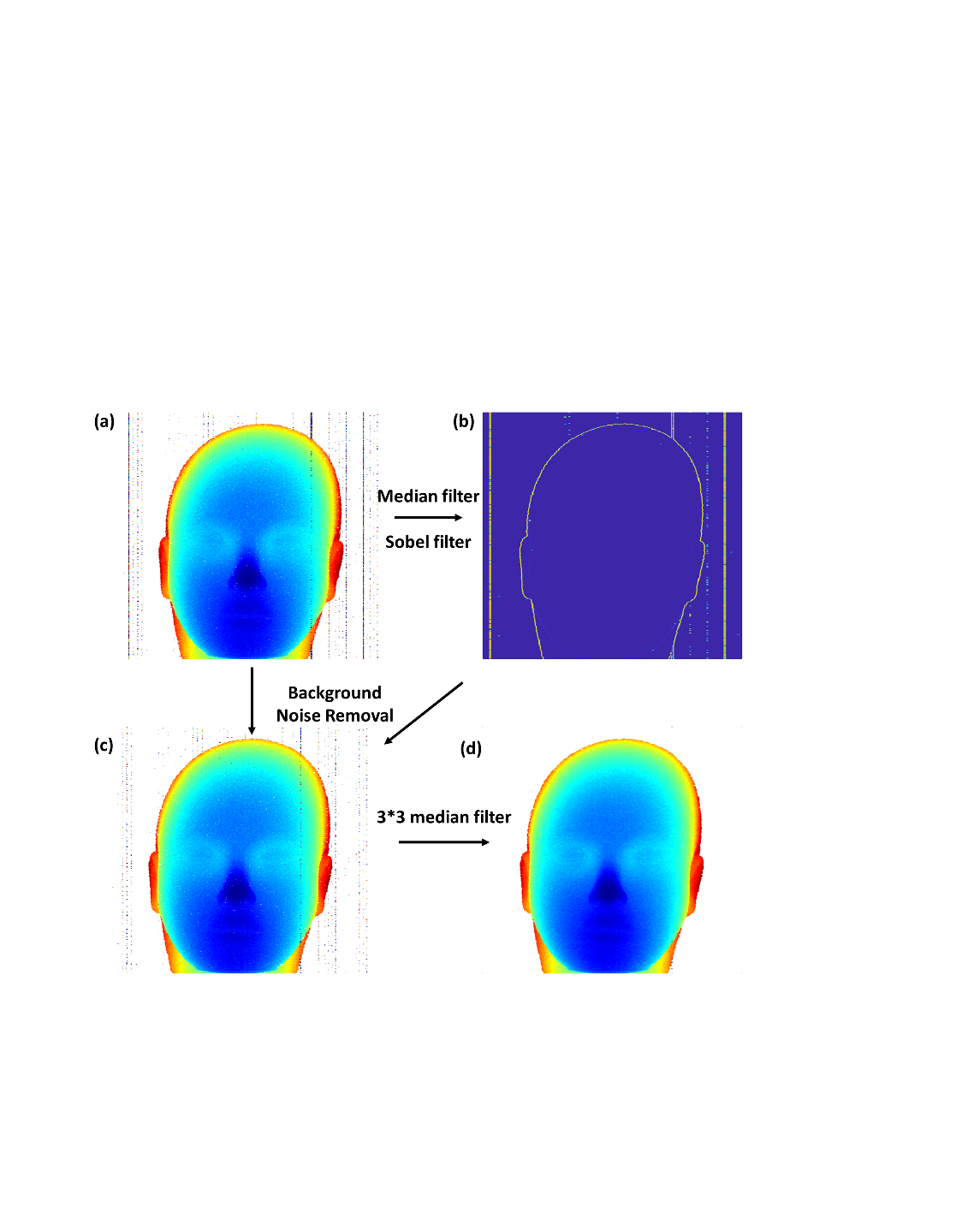


Supplement Figure 2: Image processing pipeline to remove the noise in the processed depth maps. (a) The original depth map of the mannequin head after depth localization; (b)the gradient magnitude map calculated after applying a median filter and a Sobel filter to the depth map; (c) the depth map after removing the pixels with gradient magnitudes above the certain threshold value; (d) the final processed depth map after applying a 3*3 median filter.

**Predefined threshold for depth localization**

In this section, we explored the extracted depth map at various intensity threshold levels. In the main text, we calculated the mean, $\mu_{I}$, and standard deviation, $\sigma_{I}$, of the background peak intensity across the whole FOV when no object was present, and used $\mu_{I}$ + 2* $\sigma_{I}$as our intensity threshold value for peak localization. Here, the depth maps of the mannequin head at four different intensity threshold levels, $\mu_{I}$ (Supplement Fig.3(a)), $\mu_{I}$ + $\sigma_{I}$ (Supplement Fig.3(b)), $\mu_{I}$ + 2* $\sigma_{I}$(Supplement Fig.3(c)) and $\mu_{I}$ + 4* $\sigma_{I}$ (Supplement Fig.3 (d)) were shown in Supplement Fig.3. Significant amounts of background noise were present in the depth map with threshold values of $\mu_{I}$ and $\mu_{I}$ + $\sigma_{I}$ (Supplement Fig.3(a-b)), while significantly more pixels within the sample were not identified in the depth map with $\mu_{I}$ + 4* $\sigma_{I}$ threshold value compared to the depth map with $\mu_{I}$ + 2* $\sigma_{I}$ threshold value (Supplement Fig.3(c) vs. Supplement Fig.3(d), Zoom-in view: Supplement Fig.3(e) vs. Supplement Fig.3(f)). Therefore, $\mu_{I}$ + 2* $\sigma_{I}$ appears to be a reasonable value for the depth localization of our system.

**
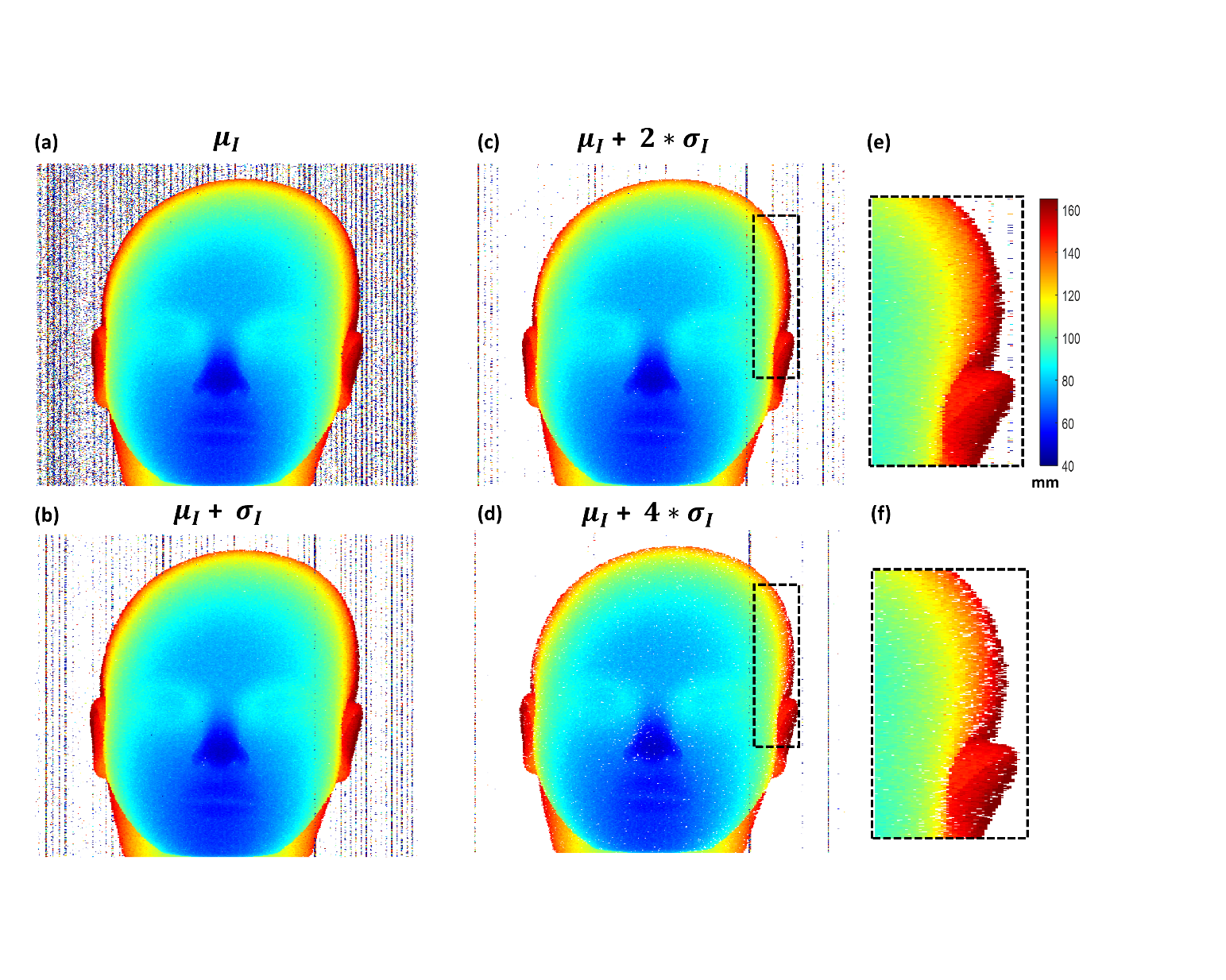
**

Supplement Figure 3: the depth maps of the mannequin head at four different intensity threshold levels. (a) $\mu_{I}$, (b) $\mu_{I}$ + $\sigma_{I}$, (c) $\mu_{I}$ + 2* $\sigma_{I}$(zoom-in view: (e)) and (d) $\mu_{I}$ + 4* $\sigma_{I}$(zoom-in view: (f)).

**Axial resolution characterization based on FWHM measurements**

To experimentally characterize the depth resolution of the system, we imaged an anodized aluminum sample, then measured the FWHM of the peak in the FFT signal for each STFT window. The averaged FWHM measured from a total of 238 STFT windows across the whole bandwidth was found to be 3.43mm, which is close to our theoretical axial resolution, 2.82mm, calculated using the coherence length equation. The sources of error here can be: (1) the anodized aluminum sample is not a perfect single reflector; (2) the sample may be slightly tilted, which can broaden the peak within each STFT window; (3) the spectral shape within each STFT is not exactly Gaussian. One representative peak signal after STFT processing is shown in Supplement Fig.4, along with its best Gaussian fit and the corresponding FWHM value.”


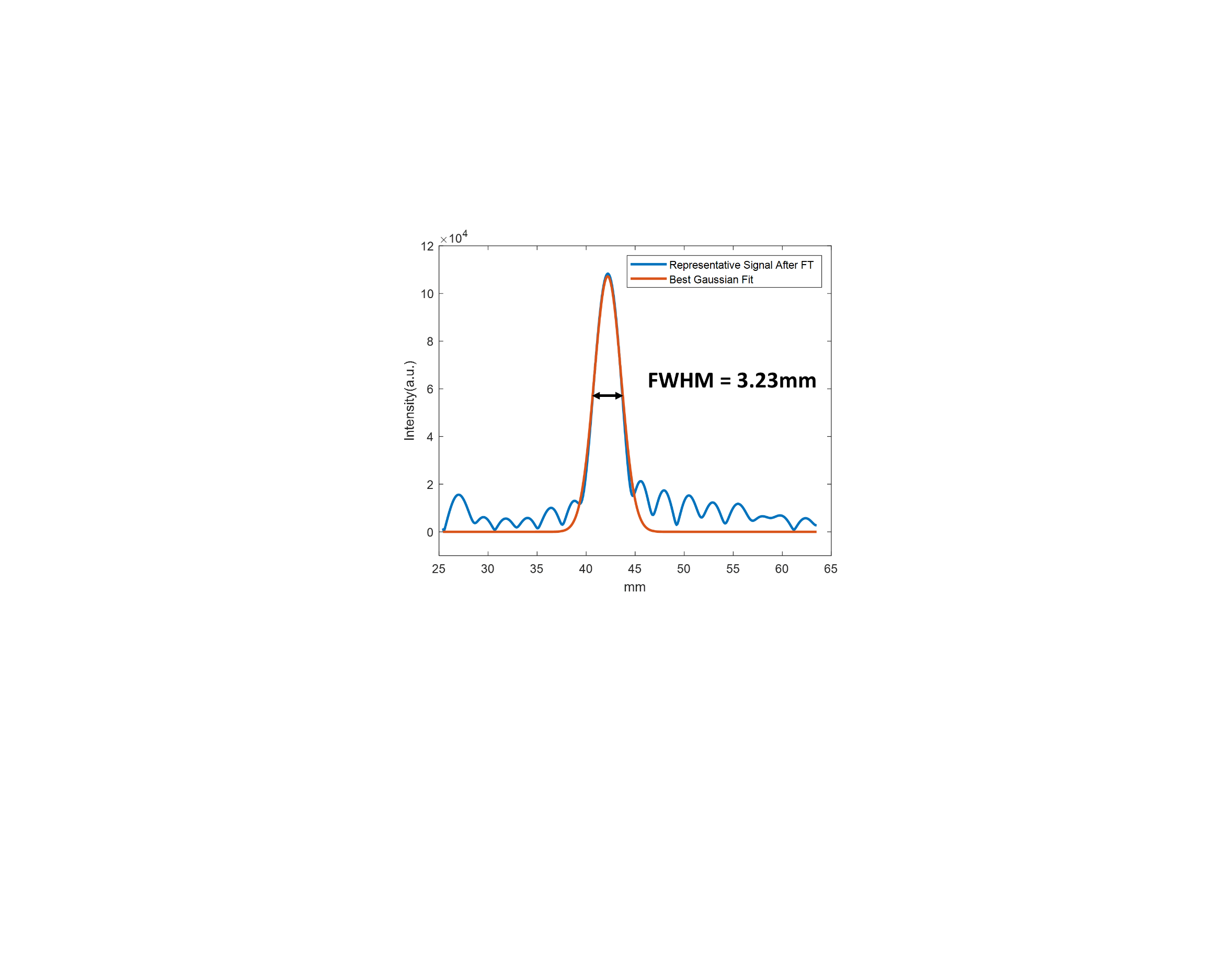


**Supplement Figure 4:** Representative peak signal of the anodized aluminum sample after STFT processing along with its best Gaussian fit and the corresponding FWHM value

**Reference**

1 Zhang, B., Zerubia, J. & Olivo-Marin, J.-C. Gaussian approximations of fluorescence microscope point-spread function models. *Appl. Opt.* **46**, 1819-1829 (2007).

2 Bonesi, M. *et al.* Akinetic all-semiconductor programmable swept-source at 1550 nm and 1310 nm with centimeters coherence length. *Opt. Express* **22**, 2632-2655 (2014).

3 Kanopoulos, N., Vasanthavada, N. & Baker, R. L. Design of an image edge detection filter using the Sobel operator. *IEEE Journal of Solid-State Circuits* **23**, 358-367 (1988).
