# Supplementary material for: Video-rate high-precision time-frequency multiplexed 3D coherent ranging
Source: Nat Commun. 2022 Mar 29;13:1476. doi: 10.1038/s41467-022-29177-9 (PMC8964719; doi:10.1038/s41467-022-29177-9)
Supplement: Supplementary file 3 — Description of Additional Supplementary Files [file 41467_2022_29177_MOESM3_ESM.pdf]

Title: Supplement Movie 1:
Description: Video of 3D surface renderings of the hand while flexing acquired with a frame rate of 33.2Hz
